# Diagnostic accuracy of circulating miRNAs to discriminate hepatocellular carcinoma from liver cirrhosis: a systematic review and meta-analysis

**DOI:** 10.3389/fmed.2024.1359414

**Published:** 2024-04-24

**Authors:** Ermiyas Alemayehu, Melaku Ashagrie Belete, Muluken Walle, Fasil Getu, Zewudu Mulatie, Mulugeta Teshome, Denekew Tenaw Anley, Daniel Gebretsadik Weldehanna, Alemu Gedefie, Hussen Ebrahim

**Affiliations:** ^1^Department of Medical Laboratory Sciences, College of Medicine and Health Sciences, Wollo University, Dessie, Ethiopia; ^2^Department of Medical Laboratory Sciences, College of Medicine and Health Sciences, Jigjiga University, Jigjiga, Ethiopia; ^3^Department of Medical Laboratory Science, Dessie Health Science College, Dessie, Ethiopia; ^4^Department of Public Health, College of Health Sciences, Debre Tabor University, Debre Tabor, Ethiopia

**Keywords:** miRNAs, non-coding RNAs, diagnostic biomarkers, hepatocellular carcinoma, HCC, liver cancer, liver cirrhosis, meta-analysis

## Abstract

**Introduction:**

Hepatocellular carcinoma (HCC) and liver cirrhosis (LC) stand as the primary causes of global mortality. Given their profound impact, the development of highly sensitive and specific circulating diagnostic markers becomes imperative to effectively identify and differentiate between cirrhosis and HCC. Accurate diagnosis is paramount in guiding appropriate therapeutic interventions. Hence, this study aimed to evaluate the potential of microRNAs (miRNAs) in discerning between HCC and LC.

**Methods:**

This study followed the Preferred Reporting Items for Systematic Review and Meta-Analysis (PRISMA) guidelines, with the protocol officially registered on PROSPERO under the reference number CRD42023417494. A thorough search across multiple databases like PubMed, Embase, Scopus, Wiley Online Library, and Science Direct was conducted to identify relevant studies published from January 1, 2018, to August 10, 2023. The included studies underwent methodological quality assessment using the Quality Assessment of Diagnostic Accuracy Studies 2 (QADAS-2) tool. The synthesis of pooled sensitivity, specificity, and other relevant diagnostic parameters employed a random-effects model and was conducted using Stata 14.0. Heterogeneity was assessed using *I*^2^ and Cochrane Q, with subsequent subgroup analysis and meta-regression performed to identify potential sources of observed heterogeneity. A sensitivity analysis was performed to assess the resilience of the findings. Furthermore, Deeks’ funnel plot was employed to evaluate publication bias.

**Results:**

In this meta-analysis, we included fifteen publications, encompassing 787 HCC patients and 784 LC patients. The combined sensitivity, specificity, positive likelihood ratio (PLR), negative likelihood ratio (NLR), diagnostic odds ratio (DOR), and area under the curve (AUC) values of miRNAs in differentiating HCC from LC were 0.84 (95% CI: 0.78–0.88), 0.79 (95% CI: 0.73–0.84), 3.9 (95% CI: 3.0–5.2), 0.21 (95% CI: 0.14–0.29), 19.44 (95% CI: 11–34), and 0.88 (95% CI: 0.85–0.91), respectively. The results of the subgroup analysis revealed that upregulated miRNA levels and miRNA assessments specifically for individuals of European descent exhibited superior diagnostic performance.

**Conclusion:**

The results of this study suggested that circulating miRNAs, especially those that are upregulated, have the potential to function as robust and promising biomarkers in the differentiation of HCC from LC.

**Systematic review registration:**

https://www.crd.york.ac.uk/prospero/display_record.php?ID=CRD42023475954.

## Introduction

Hepatocellular carcinoma (HCC) stands as the predominant form of liver cancer, commonly emerging as a consequence of liver cirrhosis (LC). Its association is notably linked to hepatitis virus infections and the presence of alcoholic or non-alcoholic fatty liver disease (1). According to the most recent global burden of disease statistics for 2020, liver cancer holds the sixth position among the most frequently diagnosed cancers and ranks as the third leading cause of cancer-related mortality worldwide. Approximately 905,700 individuals were diagnosed with liver cancer, resulting in 830,200 deaths (2).

Conversely, LC represents a late-stage scarring process wherein healthy liver tissue is replaced by nodules and scar tissue encircled by fibrous bands, stemming from prolonged liver injury and damage. The irreversible progression of cirrhosis may end in the development of HCC (3). It serves as the primary cause of liver-related deaths globally (4). In 2017, cirrhosis accounted for over 1.32 million deaths globally, with 440,000 in females and 883,000 in males, comprising 2.4% of the total global mortality for that year (5).

An association exists between liver cirrhosis and HCC, where cirrhosis may either precede the development of HCC or coexist with it (6). The diagnosis of HCC is frequently delayed as symptoms become noticeable only in the later stages or when the tumor is relatively larger. Consequently, both detection and treatment are often postponed, resulting in a diminished life expectancy for the patient (7). This delay is primarily attributed to the absence of an effective method for early diagnosis (8).

In general, the primary diagnostic pathways for HCC involve histopathological examination, blood biomarkers, and imaging techniques (9). Although serum alpha-fetoprotein (AFP) remains a widely used biomarker for HCC screening, early diagnosis, and therapeutic assessment (10), it has notable limitations, including low sensitivity and specificity. AFP can be elevated in some patients with cirrhosis or hepatic inflammation in the absence of a tumor, and it may not increase in 80% of small tumors (11, 12). Other methods, such as the gold standard liver biopsy and certain imaging platforms, face challenges like cost, invasiveness, limited availability in developing countries, and susceptibility to sampling errors and observer variations (13). Hence, developing sensitive and specific circulating diagnostic markers to identify and distinguish between cirrhosis and HCC is crucial, as the accurate diagnosis will play a pivotal role in determining the most suitable therapy.

The acknowledgment of microRNAs (miRNAs) has ushered in a new era of research focused on discovering novel non-invasive markers for cancer detection (14). Representing naturally occurring non-coding, single-stranded small RNA molecules ranging from 19 to 24 nucleotides in length (15), miRNAs play a crucial role in regulating posttranscriptional gene expression in the genome. They inhibit target genes by binding to the 3′ untranslated region (3’UTR) within messenger RNA (mRNA), leading to either mRNA degradation or the inhibition of protein translation (16, 17).

Altered miRNA expression has been observed in various cancer types, including lung, prostate, colon, breast, and liver tumors, influencing the activity of oncogenes and tumor suppressor genes and directly impacting carcinogenesis (18). In liver development, homeostasis, and pathophysiology, numerous miRNAs play essential roles (19). Due to their dysregulated expression, circulating miRNAs have been explored as potential biomarkers for cancer, including HCC, and can be identified in serum or plasma through non-invasive techniques (1). Several studies have demonstrated the potential of circulating miRNAs to differentiate between HCC and LC (20–22). Nevertheless, owing to inconsistencies observed in prior research, drawing reliable conclusions regarding the effectiveness of circulating miRNAs for distinguishing HCC from LC remains challenging. It is crucial to consider the need for aggregated data to provide a more comprehensive and conclusive assessment. Therefore, this study aimed to evaluate the discriminative potential of circulating miRNAs in distinguishing HCC from LC, utilizing recent data.

## Methods

### Study design and protocol registration

This study protocol was registered in PROSPERO (CRD42018104269) and was conducted according to the Preferred Reporting Items for Systematic Reviews and Meta-Analyses (PRISMA) guidelines (Supplementary Table S1).

### Search strategy

Electronic databases like PubMed, Embase, Scopus, Wiley Online Library, and ScienceDirect were searched to identify relevant articles reporting the diagnostic accuracy of circulating microRNA to discriminate HCC patients from LC patients from the time of inception to August 10, 2023. The following search terms were included: “circulating miRNAs” OR “circulating microRNAs” OR “circulating microRNA” OR “circulating miR*” OR “plasma miRNAs” OR “plasma microRNAs” OR plasma microRNA” OR “plasma miR*” OR “serum miRNAs” OR “serum microRNAs” OR “serum microRNA” OR “serum miR*” AND “diagnos*” AND “hepatocellular carcinoma” OR “HCC.” In addition, a manual search of relevant articles and references cited in these articles was conducted to identify all available studies. The detailed search is presented in Supplementary Table S2.

### Eligibility criteria and quality assessment

The inclusion criteria encompassed the following criteria: (1) observational study; (2) human studies used miRNAs to discriminate HCC patients from LC patients; (3) false positive (FP), true positive (TP), false negative (FN), and true negative (TN) could be derived directly or calculated from the literature; and (4) studies published since January 1, 2018 G.C. The exclusion criteria were set as follows: (1) non-human studies; (2) full-text unavailability; (3) reviews, letters to editorials or conference proceedings; and (4) studies with inadequate information regarding diagnostic performance, sensitivity or specificity.

Two investigators (ZM and AG) used the Quality Assessment of Diagnostic Accuracy Studies-2 (QUADAS-2) tool (23) to independently assess the risk of bias and clinical applicability of included studies. The tool comprised four main components: (1) patient selection, (2) the index test, (3) the reference standard, and (4) flow and timing. Bias risk was graded as high (H), low (L), or unclear (U). Disagreements were resolved by agreement between the two investigators through negotiation or by involving a third reviewer (HE).

### Study selection and data extraction

Two independent reviewers (MW and FG) systematically reviewed all studies with title and abstract for inclusion and the full text for the primary review. In cases of disagreements, the conclusion was finalized by a discussion with the third reviewer (MB). Then, data were extracted independently by two investigators (DA and EA) and from eligible studies, including first author, year of publication, country, miRNAs, type of samples, internal reference, cut-off values, sample size (HCC and LC patients), assay method, miRNAs expression, AUC with 95% confidence intervals (CIs), sensitivity, and specificity. The TP, FP, FN, and TN values were calculated using sensitivity, specificity and sample sizes.

### Statistical analysis

Stata 14.0 software was used for the meta-analysis. Furthermore, Review Manager 5.4 was used to assess the quality of the included studies. Based on the random effect model, the pooled sensitivity, specificity, DOR, PLR, NLR, and corresponding 95% confidence interval (CI) of the included literature were determined using TP, FP, FN, and TN values (24). Summary receiver operating characteristic (SROC) curves were plotted to calculate the area under the curve (AUC) to test the pooled diagnostic value of miRNAs and to assess the presence of threshold effect. Cochrane Q test and *I*^2^ statistics were used to assess the heterogeneity between studies, with *p*-value less than 0.05 for Cochran-Q test and *I*^2^ > 50%, indicating significant heterogeneity between studies (25). The potential heterogeneity sources were analyzed through meta-regression and subgroup analyses. Additionally, sensitivity analysis was conducted to check the stability of the meta-analysis results. Deek’s quantitative funnel plot was used to assess the publication bias between studies. *p* < 0.05 denotes for the statistical significance. In addition, the clinical value of circulating miRNAs to discriminate hepatocellular carcinoma patients from liver cirrhosis patients was evaluated using Fagan’s plots.

## Results

### Search results, description, and quality assessment of the included studies

**Figure 1:**
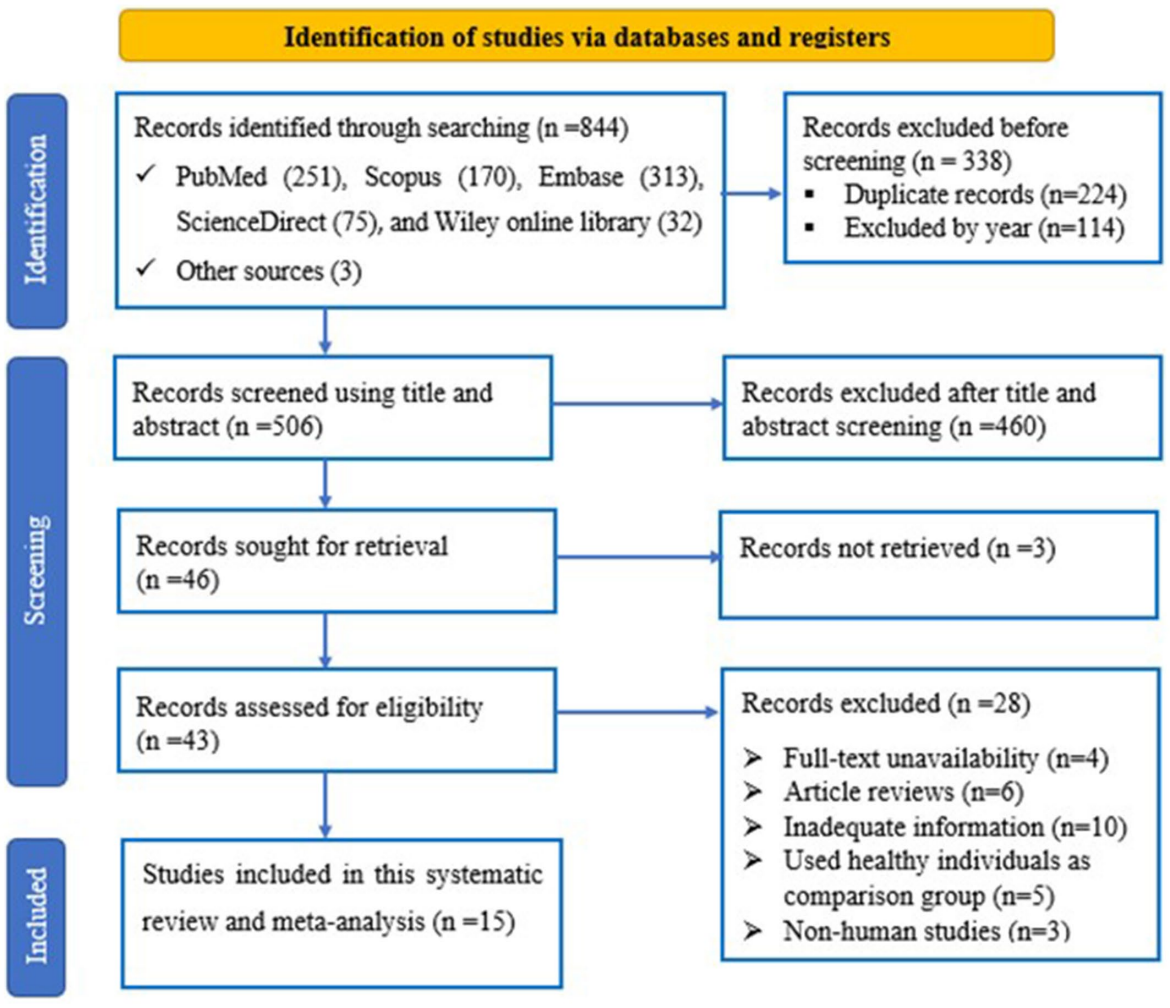
PRISMA flowchart depicting the article selection process.

The flowchart represents the search and selection strategy for the study. The initial search resulted in a total of 844 studies, consisting of PubMed (*n* = 251), Scopus (*n* = 170), Embase (*n* = 313), ScienceDirect (*n* = 75) and Wiley online library (*n* = 32) articles, together with articles identified through relevant bibliography search (*n* = 3). 224 and 114 articles were excluded because of duplication and year, respectively. Furthermore, 460 articles were excluded because of title and abstract screening criteria. The full-text of the remaining 43 articles was reviewed. In addition, 28 articles were excluded from full-text review and finally 15 studies were considered for this meta-analysis (Figure 1).

**Figure 2:**
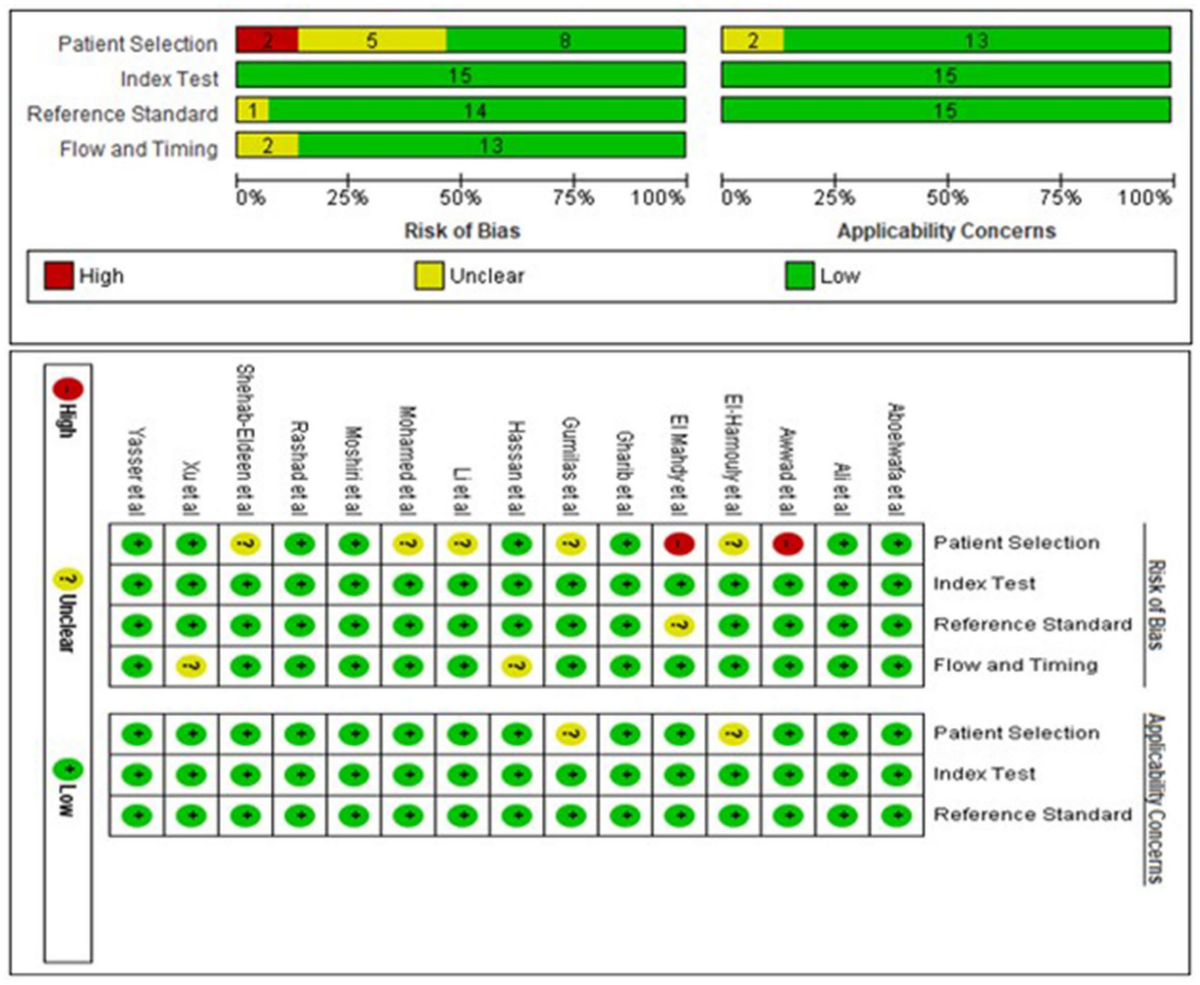
Risk of bias assessment of studies using QUADAS-2.

A total of 1,571 participants, including 787 HCC patients and 784 LC patients from 15 studies were included in the data analysis. The sample size ranged from 16 to 100 in the HCC cohort, and 20 to 100 in the LC cohort. Additionally, two studies were from China (22, 26), eleven studies were from Egypt (21, 27–36), one study was from Indonesia (20) and one study was from Italy (37). Seven studies used plasma samples, whereas four studies used serum samples for miRNA quantification. MiRNAs were measured using quantitative reverse transcription polymerase chain reaction (qRT-PCR). Furthermore, fifteen miRNAs were upregulated and ten miRNAs were downregulated. The baseline characteristics of the included studies are summarized in Table 1. Based on the Quality Assessment of Diagnostic Accuracy Studies 2 evaluation tool, the RevMan 5.4 software was used to evaluate the quality of the included studies. The results are shown in Figure 2.

**Table 1:** Characteristics of included circulating miRNA studies in this meta-analysis.

Authors	Year	County	miRNAs	Expression	Specimen	Method	Reference	Participants				Cut-off	Sen (%)	Spe (%)	AUC
								Case	No	Control	No				
Xu et al. (26)	2018	China	miR-125b	Down	Serum	qRT-PCR	U6	HBV-HCC	100	LC	100	2.91	78	96	0.91
Li et al. (22)	2019	China	miR-122	Up	Serum	qPCR	cel-miR-39	HCC	47	LC	35	N/A	97.23	79.41	0.900
Rashad et al. (27)	2018	Egypt	miR-27a	Down	Serum	qRT-PCR	SNORD68	HCV-HCC	51	LC	39	2.38	96.7	71.7	0.897
Rashad et al. (27)	2018	Egypt	miR-18b	Down	Serum	qRT-PCR	SNORD68	HCV-HCC	51	LC	39	0.62	75.6	46.7	0.723
Rashad et al. (27)	2018	Egypt	miR-27a, 18b	N/A	Serum	qRT-PCR	SNORD68	HCV-HCC	51	LC	39	NA	91.1	71.7	0.821
El-Mahdy et al. (28)	2019	Egypt	miR-215	Down	Plasma	qRT-PCR	RNU6	HCC	60	LC	75	1.90	78.3	88	0.87
Ali et al. (29)	2019	Egypt	miR-215	Up	Serum	qRT-PCR	SNORD68_11	HCV-HCC	60	LC	60	4.17	97.14	91	0.997
El-Hamouly et al. (30)	2019	Egypt	miR-301	Up	Plasma	qRT-PCR	U6	HCV-HCC	42	LC	48	9.91	78.57	89.58	0.89
Hassan et al. (31)	2019	Egypt	miR-483-5p	Up	Serum	qRT-PCR	SNORD 68	HCV-HCC	20	LC	20	3.89	100	75	0.907
Hassan et al. (31)	2019	Egypt	miR-133a	Up	Serum	qRT-PCR	SNORD 68	HCV-HCC	20	LC	20	4.79	70	90	0.84
Shehab-Eldeen et al. (21)	2019	Egypt	miR-122	Down	Serum	qRT-PCR	U6	HCV-HCC	20	LC	20	1.19	95	81	0.93
Shehab-Eldeen et al. (21)	2019	Egypt	miR-224	Up	Serum	qRT-PCR	U6	HCV-HCC	20	LC	20	0.99	85	79	0.77
Mohamed et al. (32)	2020	Egypt	miR-155	Up	Serum	qRT-PCR	RNU6B	HCV-HCC	80	LC	80	4.30	80	62.5	0.743
Mohamed et al. (32)	2020	Egypt	miR-665	Up	Serum	qRT-PCR	RNU6B	HCV-HCC	80	LC	80	2.23	92.5	86.3	0.930
Aboelwafa et al. (33)	2021	Egypt	miR-331-3p	Up	Plasma	qRT-PCR	RNU6	HCC	50	LC	100	2.18	66	61	0.703
Aboelwafa et al. (33)	2021	Egypt	miR-23-3p	Down	Plasma	qRT-PCR	RNU6	HCC	50	LC	100	0.36	80	74	0.781
Awwad et al. (34)	2021	Egypt	miR-221	Up	Plasma	qRT-PCR	RNU6B	HCV-HCC	20	LC	20	1.0317	85	55	0.758
Yasser et al. (35)	2021	Egypt	miR-221	Up	Plasma	qRT-PCR	miR-39	HCV-HCC	40	LC	39	0.36	72.22	50.00	0.644
Yasser et al. (35)	2021	Egypt	miR-542	Down	Plasma	qRT-PCR	miR-39	HCV-HCC	40	LC	39	1.08	65.71	54.84	0.640
Gharib et al. (36)	2022	Egypt	miR-96-5p	Up	Serum	qRT-PCR	miR-16	HCV-HCC	55	LC	55	1.44	69.1	85.5	0.82
Gharib et al. (36)	2022	Egypt	miR-99a-5p	Down	Serum	qRT-PCR	miR-16	HCV-HCC	55	LC	55	0.76	70.9	90.9	0.86
Gumilas et al. (20)	2022	Indonesia	miR-122	Down	Plasma	qRT-PCR	miR-16	HCC	27	LC	66	9.11	37.04	75.76	0.538
Gumilas et al. (20)	2022	Indonesia	miR-150	Down	Plasma	qRT-PCR	miR-16	HCC	27	LC	66	1.47	62.96	78.79	0.676
Moshiri et al. (37)	2018	Italy	miR-101-3p	Up	Plasma	ddPCR	NA	HCC	16	LC	27	NA	86.7	80.0	0.91
Moshiri et al. (37)	2018	Italy	miR-1246	Up	Plasma	ddPCR	NA	HCC	16	LC	27	NA	86.7	84.6	0.97
Moshiri et al. (37)	2018	Italy	miR-106b-3p	Up	Plasma	ddPCR	NA	HCC	16	LC	27	NA	90.9	72.2	0.91
Moshiri et al. (37)	2018	Italy	miR-101-3p, 1,246,106b-3p	NA	Plasma	ddPCR	NA	HCC	16	LC	27	NA	100.0	92.9	0.99

HCC, hepatocellular carcinoma; HCV, hepatitis C virus; HBV, hepatitis B virus; NA, not available; qRT-PCR, quantitative real-time polymerase chain reaction; ddPCR, digital droplet polymerase chain reaction.

### Diagnostic accuracy of miRNAs in distinguishing HCC patients from LC patients

**Figure 3:**
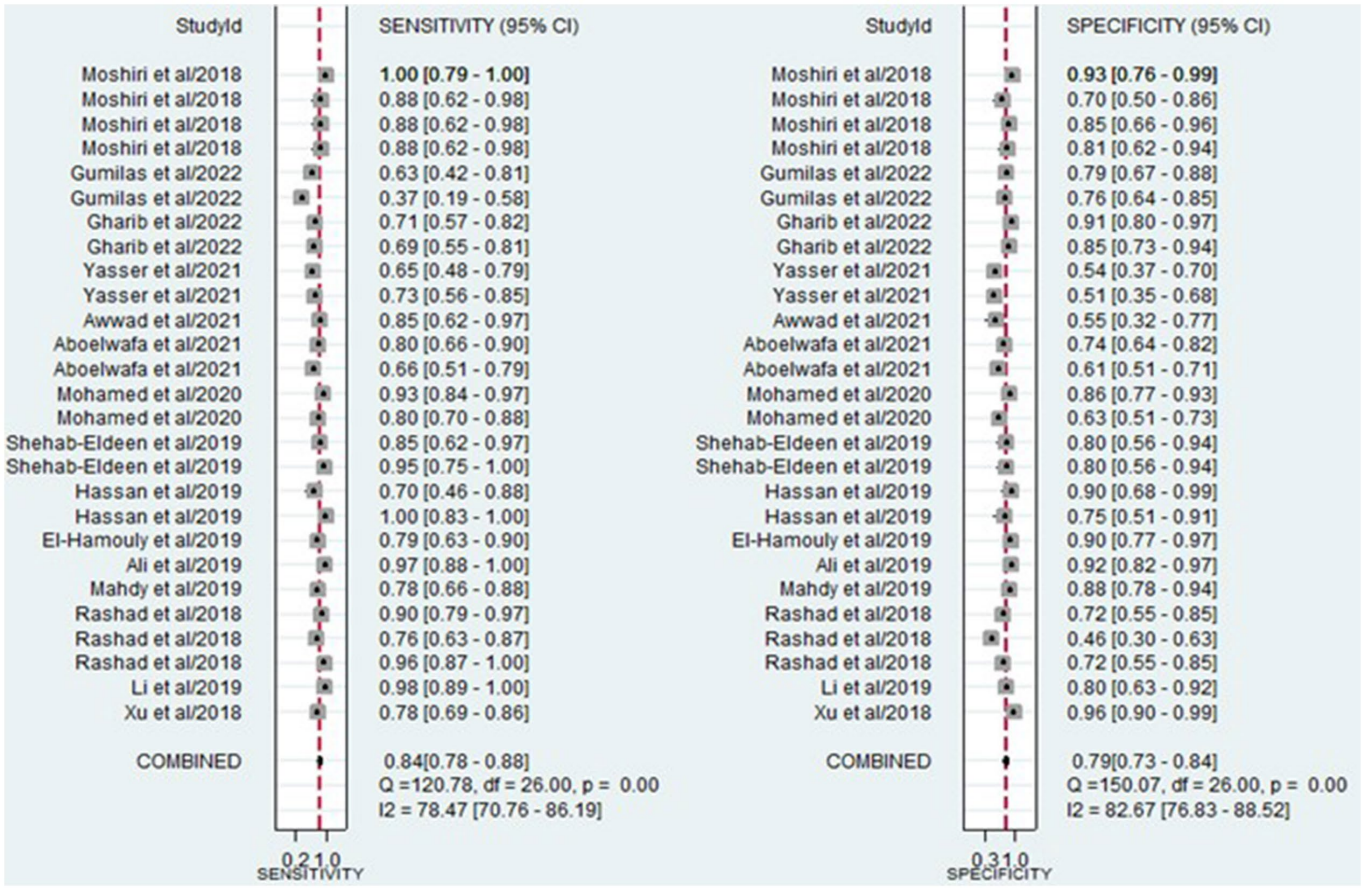
Forest plot illustrating the sensitivity and specificity of miRNAs in the discrimination of HCC and LC patients.

**Figure 4:**
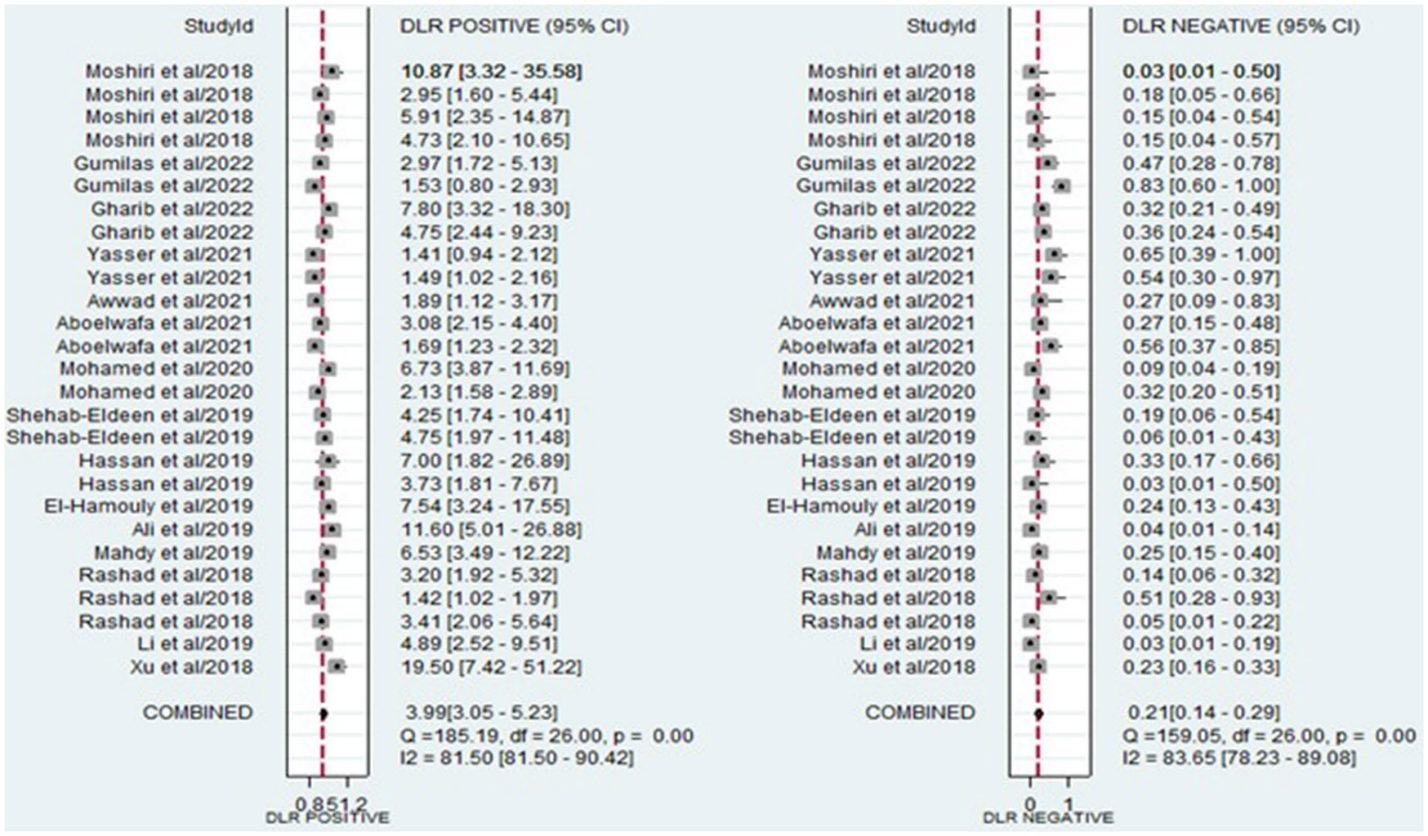
Forest plot illustrating the PLR and NLR of miRNAs in the discrimination of HCC and LC patients.

**Figure 5:**
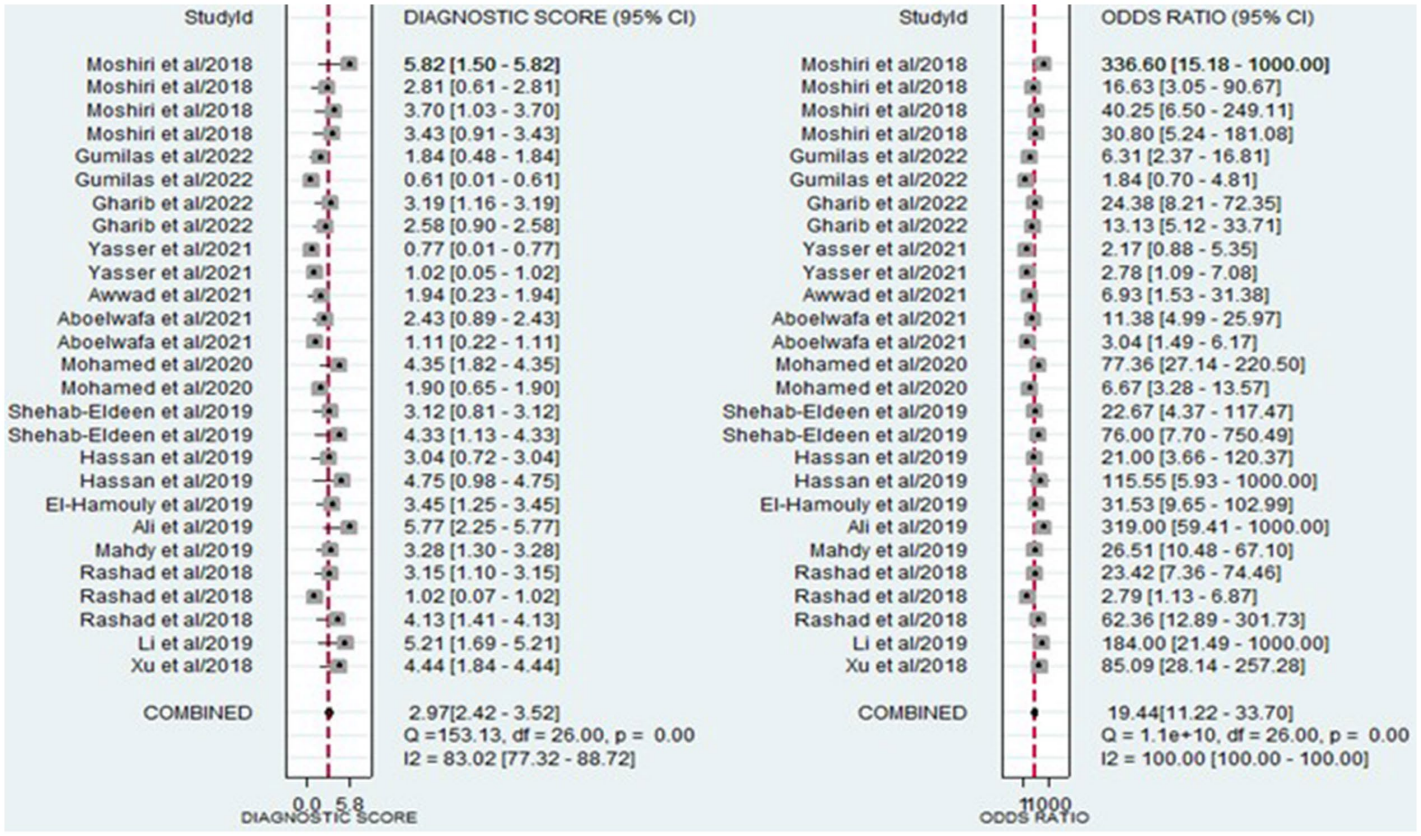
Forest plot illustrating the DOR of miRNAs in the discrimination of HCC and LC patients.

**Figure 6:**
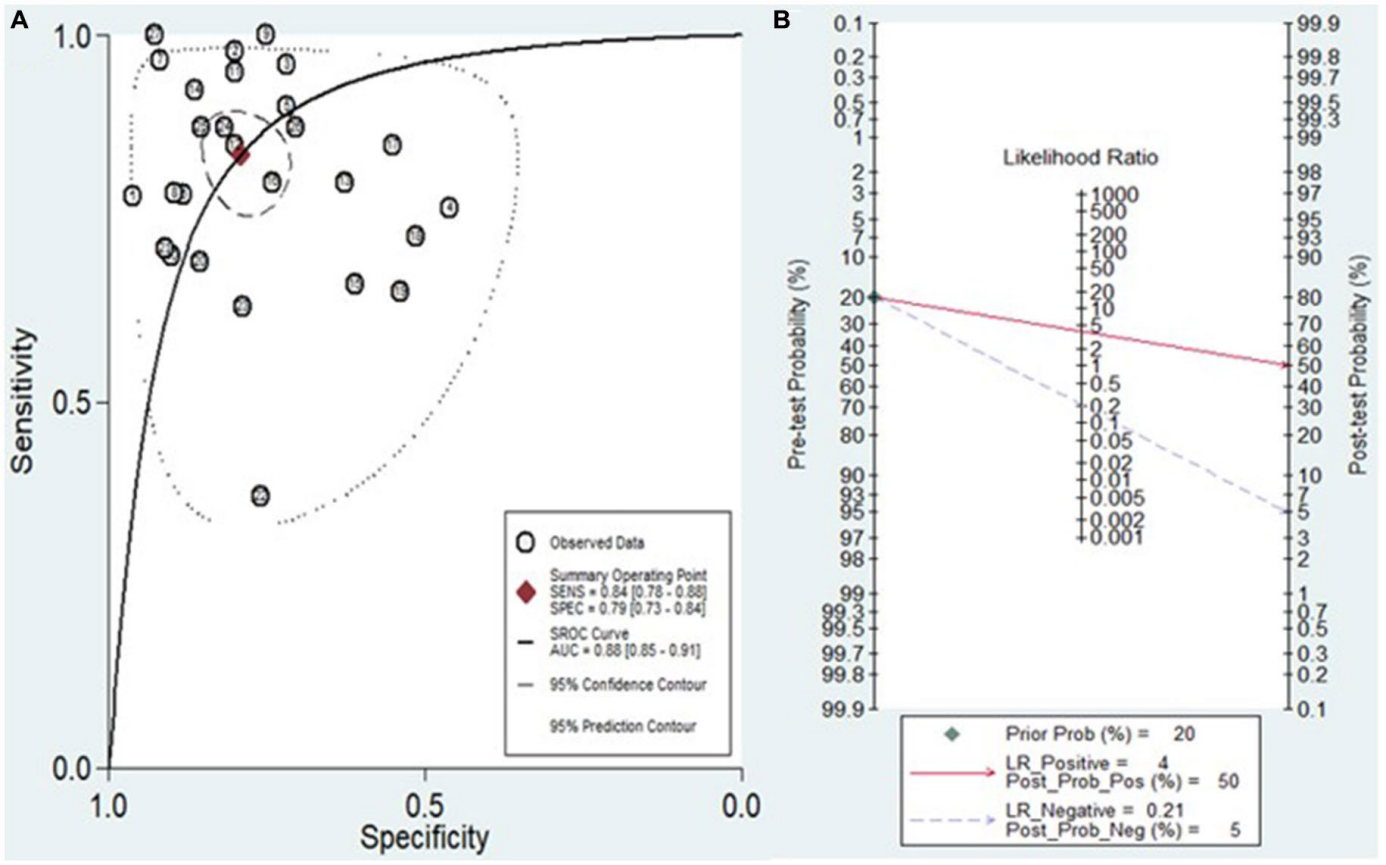
**(A)** Diagram of SROC curves illustrating the diagnostic performance of miRNAs in discriminating between HCC and LC patients. **(B)** The Fagan nomogram illustrates the capacity of miRNA testing to either confirm or exclude HCC in patients.

The analysis involved summarizing estimates of miRNA diagnostic accuracy in distinguishing between HCC and LC. The combined sensitivity and specificity for diagnosing HCC with miRNAs were 0.84 (95% CI: 0.78–0.88) and 0.79 (95% CI: 0.73–0.84), respectively (Figure 3). Notably, there was substantial heterogeneity observed among the studies, with an *I*^2^ value of 78.47% for sensitivity and 82.67% for specificity. Additionally, the pooled PLR, NLR, and DOR were 3.9 (95% CI: 3.0–5.2), 0.21 (95% CI: 0.14–0.29), and 19.44 (95% CI: 11–34), respectively (Figures 4, 5). The shape of the SROC curve did not exhibit the typical “shoulder-arm-like” pattern, suggesting that there is no evident threshold effect in this study. The AUC was 0.88 (95% CI: 0.85–0.91), indicating that overall, miRNAs demonstrate exceptional diagnostic accuracy (Figure 6A). Moreover, the Fagan nomogram was utilized to evaluate the efficacy of miRNA testing in confirming or ruling out the presence of HCC in patients. The results revealed that, when the pre-test probability was set at 20%, the post-test probabilities for the PLR and NLR were 50 and 5%, respectively. Consequently, miRNA testing assumes a crucial role in the initial screening of individuals with HCC (Figure 6B).

### Subgroup analyses and meta-regression

In our pursuit to identify sources of heterogeneity among the studies, we conducted both subgroup analysis and meta-regression analysis. This involved categorizing studies based on ethnicity, sample source, regulation mode, miRNA profiling, sample size, cut-off value presence, and types of HCC.

During the subgroup analysis (Table 2), we observed that studies conducted on the European population exhibited higher overall diagnostic accuracy compared to those carried out on the Asian and African populations. The combined diagnostic values, along with their 95% confidence intervals, were reported as follows: sensitivity 0.91 (0.80–0.96), specificity 0.83 (0.72–0.90), PLR 5.3 (3.1–9.2), NLR 0.11 (0.04–0.27), DOR 49 (14–173), and an AUC of 0.94 (0.91–0.96). Additionally, individual miRNAs demonstrated the following aggregated diagnostic values for distinguishing between HCC and LC: sensitivity 0.83 (0.76–0.87), specificity 0.79 (0.73–0.84), PLR 3.9 (2.9–5.1), NLR 0.22 (0.16–0.31), DOR 17 (10–30), and an AUC of 0.87 (0.84–0.90). In contrast to downregulated miRNAs, upregulated miRNAs demonstrated superior overall diagnostic accuracy, with a sensitivity of 0.86 (0.79–0.91), specificity of 0.78 (0.71–0.84), PLR of 3.9 (2.8–5.5), NLR of 0.18 (0.12–0.28), DOR of 22 (11–44), and an AUC of 0.89 (0.86–0.91). Moreover, studies not reporting their cut-off values showed superior diagnostic accuracy compared to studies reporting their cut-off values, as indicated by sensitivity 0.93 (0.87–0.97), specificity 0.80 (0.72–0.86), PLR 4.7 (3.3–6.6), NLR 0.09 (0.04–0.17), DOR 54 (22–135), and an AUC of 0.93 (0.90–0.95).

**Table 2:** Subgroup analysis of the diagnostic value of miRNAs in discriminating between HCC patients and LC patients.

Subgroup	No of studies	Sensitivity (95% CI)	Specificity (95% CI)	PLR (95% CI)	NLR (95% CI)	DOR (95% CI)	AUC (95% CI)
Ethnicity							
African	19	0.83 (0.77, 0.88)	0.76 (0.69, 0.82)	3.5 (2.6, 4.8)	0.22 (0.16, 0.31)	16 (9, 29)	0.87 (0.84–0.90)
Asian	4	0.76 (0.43, 0.93)	0.85 (0.73, 0.92)	5.1 (2.3, 11.3)	0.28 (0.09, 0.88)	18 (3, 109)	0.88 (0.85–0.91)
European	4	0.91 (0.80, 0.96)	0.83 (0.72, 0.90)	5.3 (3.1, 9.2)	0.11 (0.04, 0.27)	49 (14, 173)	0.94 (0.91–0.96)
Sample							
Serum	14	0.88 (0.81, 0.93)	0.82 (0.74, 0.88)	4.9 (3.3, 7.1)	0.14 (0.09, 0.24)	34 (17, 69)	0.83 (0.74–0.88)
Plasma	13	0.77 (0.68, 0.84)	0.76 (0.68, 0.83)	3.2 (2.2, 4.6)	0.31 (0.21, 0.45)	10 (5, 21)	0.83 (0.79–0.86)
Regulation							
Up	15	0.86 (0.79, 0.91)	0.78 (0.71, 0.84)	3.9 (2.8, 5.5)	0.18 (0.12, 0.28)	22 (11, 44)	0.89 (0.86–0.91)
Down	10	0.77 (0.65, 0.85)	0.79 (0.68, 0.87)	3.6 (2.3, 5.9)	0.30 (0.19, 0.46)	12 (5, 28)	0.85 (0.81–0.88)
miRNAs profile							
Single	25	0.83 (0.76, 0.87)	0.79 (0.73, 0.84)	3.9 (2.9, 5.1)	0.22 (0.16, 0.31)	17 (10, 30)	0.87 (0.84–0.90)
Cluster	2	-	-	-	-	-	-
Sample size							
Small (*n* < 100)	18	0.86 (0.77, 0.92)	0.75 (0.68, 0.81)	3.5 (2.6, 4.6)	0.19 (0.11, 0.32)	19 (9, 39)	0.86 (0.83–0.89)
Large (*n* ≥ 100)	9	0.81 (0.73, 0.87)	0.84 (0.75, 0.91)	5.2 (3.1, 8.7)	0.23 (0.15, 0.34)	23 (10, 52)	0.89 (0.86–0.92)
Cut-off value							
With cut-off value	21	0.80 (0.73, 0.86)	0.78 (0.71, 0.84)	3.7 (2.7, 5.1)	0.25 (0.18, 0.35)	15 (8, 27)	0.86 (0.83–0.89)
Without cut-off value	6	0.93 (0.87, 0.97)	0.80 (0.72, 0.86)	4.7 (3.3, 6.6)	0.09 (0.04, 0.17)	54 (22, 135)	0.93 (0.90–0.95)
Types of HCC							
HCV related HCC	16	0.85 (0.78, 0.90)	0.76 (0.68, 0.83)	3.6 (2.6, 5.1)	0.20 (0.13, 0.30)	18 (9, 36)	0.88 (0.85–0.91)
HCC	10	0.83 (0.68, 0.92)	0.79 (0.73, 0.85)	4.0 (2.8, 5.7)	0.21 (0.11, 0.44)	19 (7, 51)	0.86 (0.82–0.88)

HCC, hepatocellular carcinoma; HCV, hepatitis C virus.

**Figure 7:**
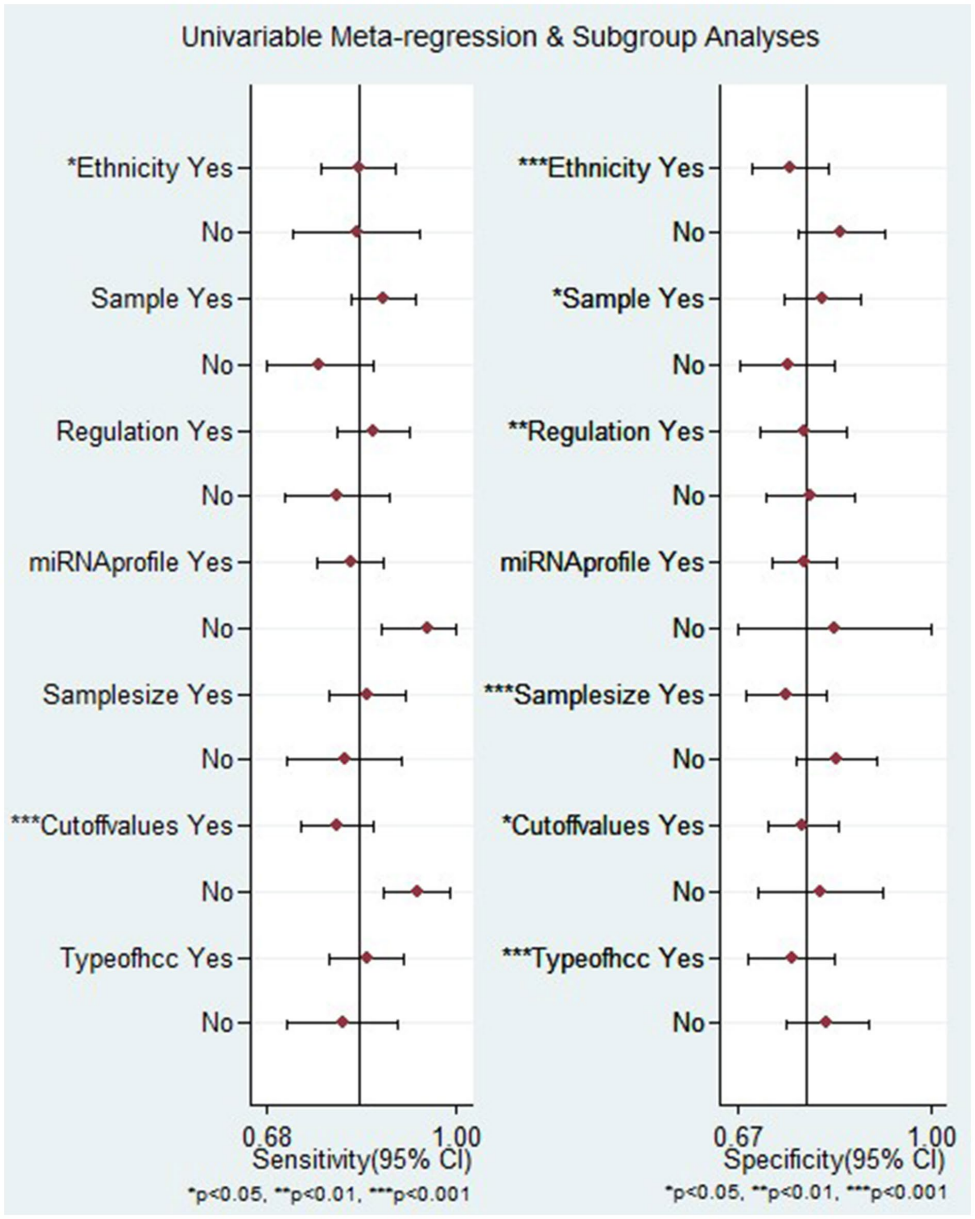
Meta-regression analysis examining the sensitivity and specificity of miRNAs in discriminating between HCC and LC patients.

Besides, the meta-regression analysis indicated that heterogeneity in sensitivity among the studies could be attributed to ethnicity and the setting of cut-off values. Conversely, factors such as ethnicity, sample source, regulation mode, sample size, cut-off value setting, and types of HCC were identified as reasons for heterogeneity in specificity among the studies (*p* < 0.05) (Figure 7).

### Sensitivity analyses and publication bias

**Figure 8:**
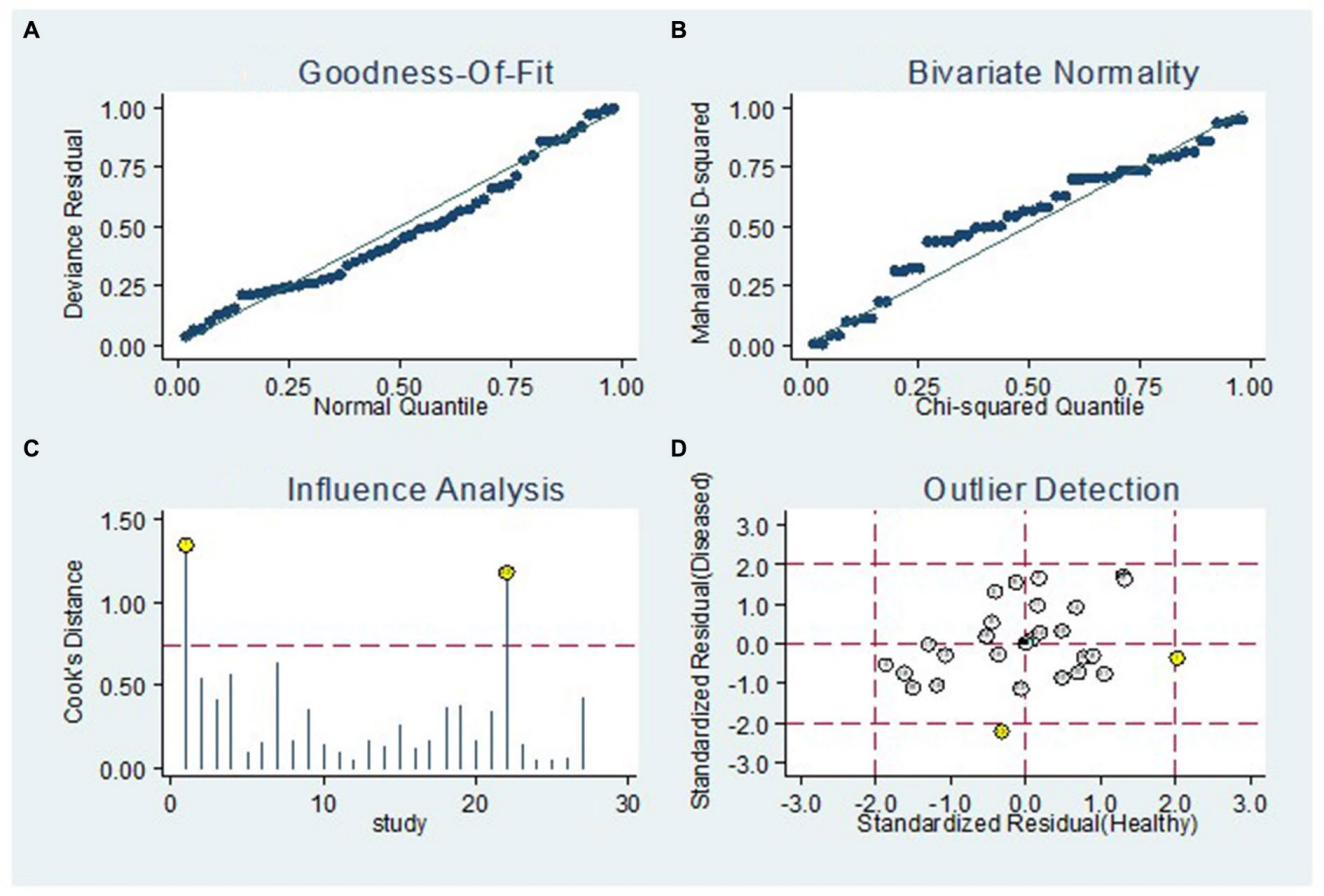
Sensitivity analysis. The diagram shows the **(A)** goodness-of-fit, **(B)** bivariate normality, **(C)** influence and **(D)** outlier detection analyses.

**Figure 9:**
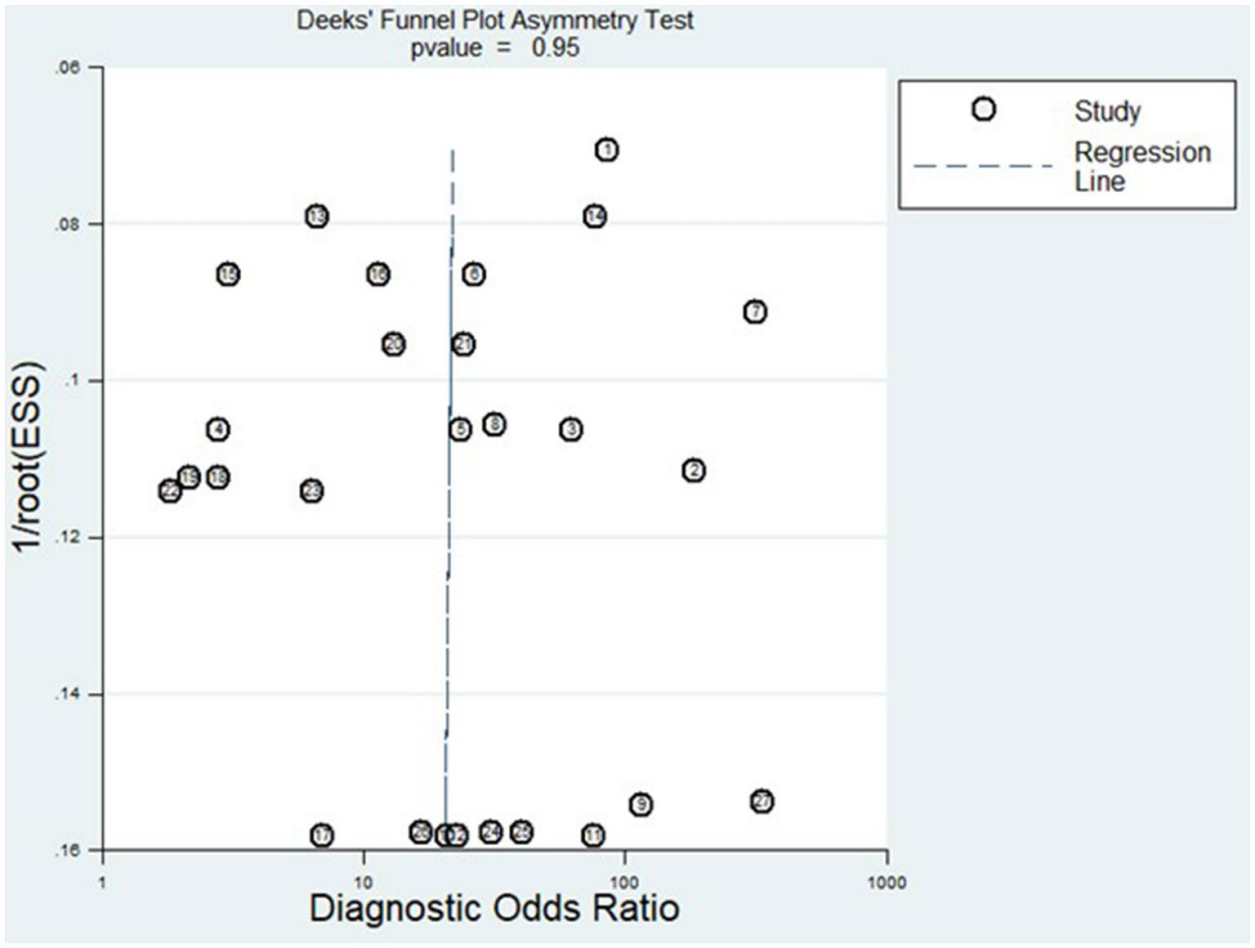
Deek’s funnel plot for publication bias analysis.

Figure 8 displays the outcomes of the sensitivity analysis. The goodness-of-fit and bivariate normal analysis, depicted in Figures 8A,B, affirm the robustness of the random effects model for meta-analysis. Furthermore, outlier detection points to two studies conducted by Xu et al. (miR-125b) (26) and Gumilas et al. (miR-122) (20) as potential sources of heterogeneity (Figure 8D). After removing these studies, the overall sensitivity remained unchanged at 0.85 (0.79–0.89), along with a specificity of 0.78 (0.72–0.83), PLR of 3.8 (3.0–5.0), NLR of 0.19 (0.14–0.27), DOR of 20 (11–34), and an AUC of 0.88 (0.85–0.91). This indicates that the sensitivity of the included studies was low, and the results became more robust and credible after excluding the identified outliers. Additionally, Figure 9 illustrates the Deeks’ funnel plot, serving as an assessment of publication bias. The *p*-value of 0.95 obtained from the analysis indicates the absence of significant publication bias in the included studies.

## Discussion

Hepatocellular carcinoma ranks as the fourth major contributor to cancer-related deaths globally and stands as a prominent cause of mortality in individuals with cirrhosis (38). The definitive diagnosis of HCC is typically differentiated from cirrhosis through the use of advanced imaging techniques, including CT scans and MRI. This distinction relies on identifying enhancement patterns in the hepatic arterial phase (HAP) images (39). Liver biopsy is used for confirmation of diagnosis or exclusion of other lesions that may mimic HCC. Diagnosing HCC is often challenging as it tends to be identified after the onset of clinical deterioration. The silent and asymptomatic growth of HCC makes it difficult to detect in its early stages (40).

The identification of early-stage HCC is crucial for initiating aggressive intervention and improving overall survival rates (41). Hence, there is a critical need to identify a more precise and advanced non-invasive biomarker for the early diagnosis of HCC, enabling the differentiation of target groups from those with similar presentations with high sensitivity and specificity. Recent evidence has indicated that abnormal miRNA profiles are associated with the development, progression, and prognosis of various human cancers (42). Consequently, miRNA has garnered significant attention from experts in the diagnosis of HCC due to its notable specificity, repeatability, and accuracy (43). However, their stability as clinical biomarkers warrants careful consideration due to variations in sample processing conditions, methodology, and biological material sources (44, 45). While RNA molecules are generally unstable, prior research indicates that miRNAs exhibit exceptional stability in plasma and serum, demonstrating resistance to RNase activity, extreme pH conditions, and multiple freeze–thaw cycles (46, 47). Nonetheless, not all miRNAs maintain stability; they exhibit diverse stability profiles influenced by factors such as sequence, secondary structure, and associations with proteins or extracellular vesicles (48). This variability poses challenges in developing robust diagnostic assays, as unstable miRNAs can yield inconsistent results, impacting test reliability and reproducibility. Therefore, identifying stable miRNA biomarkers using longitudinal studies is crucial for enhancing diagnostic accuracy. Despite previous studies recommending miRNA for distinguishing HCC from liver cirrhosis, there is limited consistency among these studies, and the findings remain inconclusive. Therefore, we conducted a systematic review and meta-analysis to assess the potential of miRNAs in distinguishing between HCC and LC patients.

This study incorporated data from 15 research articles covering 27 studies, involving a total of 787 HCC patients and 784 LC patients. The overall summary estimate revealed that the pooled sensitivity and specificity of circulating miRNAs in distinguishing HCC from LC were 0.84 (95% CI: 0.78–0.88) and 0.79 (95% CI: 0.73–0.84), respectively. Additionally, the pooled PLR, NLR, DOR, and AUC from SROC were 3.9 (95% CI: 3.0–5.2), 0.21 (95% CI: 0.14–0.29), 19.44 (95% CI: 11–34), and 0.88 (95% CI: 0.85–0.91), respectively. The combined PLR of 3.9 suggests that a positive miRNA test is associated with a 3.9-fold increase in the likelihood of diagnosing HCC. Furthermore, the NLR of 0.21 indicates a 79% increase in the probability of diagnosing HCC with a negative miRNA test. Furthermore, the pooled DOR of 19.44 (greater than 1) and AUC value of 0.88 emphasize the robust diagnostic capacity of miRNAs for discriminating between HCC and LC patients. Considering all the diagnostic values collectively, these findings strongly suggest that miRNAs have the potential to function as diagnostic markers for distinguishing between HCC and LC patients. Similarly, other meta-analyses have explored the diagnostic biomarker potential of circulating miRNAs in blood for various conditions, including Leukemia (49), osteosarcoma (43), cervical intraepithelial neoplasia (50), bladder cancer (51), and ovarian cancer (52). This phenomenon may be attributed to the capacity of miRNAs to play a role in hematopoietic differentiation and modulate the expression of oncogenes or tumor suppressor genes (53).

Several meta-analyses have delved into the diagnostic utility of biomarkers like serum alpha-fetoprotein (AFP), protein induced by vitamin K absence or antagonist II (PIVKA II), and osteopontin (OPN) in distinguishing patients with and without HCC (54–56). Notably, Jang et al. found combined AUC values of 0.786 (95% CI 0.740–0.831) for AFP, 0.729 (95% CI 0.680–0.779) for PIVKA-II, and 0.660 (95% CI 0.606–0.713) for OPN in distinguishing patients with HCC from those with LC, signifying moderate diagnostic accuracy (56). In contrast, our investigation highlighted miRNAs as promising diagnostic markers, boasting an AUC of 0.88 (95% CI: 0.85–0.91), implying potentially superior discriminatory ability between HCC and LC patients. This significant disparity needs deeper exploration into the underlying biological mechanisms driving miRNA’s enhanced diagnostic potential compared to traditional biomarkers. Moreover, it emphasizes the critical need for stringent validation studies to ascertain the reliability and reproducibility of miRNA-based diagnostic methodologies. Such efforts are pivotal for advancing the clinical translation of miRNA-based diagnostics, potentially revolutionizing HCC diagnosis and patient care.

It is important to note that there was observed heterogeneity among the studies included in this study. Therefore, the impact of these confounding factors was investigated through meta-regression and subgroup analyses. The subgroup analysis by ethnicity indicated that studies conducted on the European population showed higher overall diagnostic accuracy compared to those carried out on the Asian and African populations. The pooled AUC was 0.94. The finding is supported by a meta-analysis conducted by Wu et al. (57). The discrepancy in overall diagnostic accuracy observed between studies may stem from a combination of genetic, environmental, and disease-related factors. Additionally, the subgroup analysis by miRNAs expression, upregulated miRNAs had favorable diagnostic efficacy compared to downregulated miRNAs in differentiating HCC from LC, with an AUC of 0.89. The result is in line with other meta-analyses (57, 58). This may be attributed to their heightened sensitivity as markers of abnormal cell growth and proliferation. Additionally, their frequent association with an oncogenic role reflects the activation of tumorigenic pathways, thereby enhancing their effectiveness in detecting cancerous cells.

Subsequently, the diagnostic efficacy of miRNAs based on sample type was explored, revealing that serum-derived miRNAs exhibited relatively similar diagnostic performance when compared to plasma-derived miRNAs, with AUCs of 0.83 (0.74–0.88) and 0.83 (0.79–0.86), respectively. Thus, the detection of miRNAs in the blood using both serum miRNA assays and plasma miRNA assays is equally deemed useful as noninvasive biological methods for the early diagnosis of HCC.

On the other hand, the subgroup analysis by cut-off value showed that studies not reporting their cut-off values demonstrated superior diagnostic accuracy compared to studies with established cut-off values, as evidenced by an AUC of 0.93. This may result from differences in the number of studies and the absence of standardized cut-off values, which could introduce heterogeneity and potentially impact the overall diagnostic accuracy.

Most studies incorporated in this meta-analysis employed qRT-PCR for the detection of circulating miRNA. This method emerges as the optimal choice for future applications due to its high sensitivity in identifying low copies of miRNAs in serum samples, a crucial factor for routine testing in clinical settings (59). On the other hand, the studies analyzed in this context employed various endogenous controls, potentially contributing to data heterogeneity. The absence of a universally accepted housekeeping control for miRNA, coupled with the ongoing controversy in selecting an appropriate reference (60), complicates the standardization process.

The meta-regression analysis indicated that heterogeneity in sensitivity among the studies could be attributed to ethnicity and the setting of cut-off values. Conversely, factors such as ethnicity, sample source, regulation mode, sample size, and types of HCC were identified as reasons for heterogeneity in specificity among the studies. The potential explanation might be that different ethnicities living in diverse environments and possessing varying genetic backgrounds, lifestyles, and dietary habits yield distinct miRNA expression profiles (51).

In addition, the Fagan nomogram outcomes suggest that with a pre-test probability set at 20%, a positive miRNA test result (PLR of 50%) substantially increases the likelihood of precise identification of HCC patients from LC patients. Conversely, a negative result (NLR of 5%) significantly diminishes the probability of misdiagnosing HCC patients from those with LC. These results emphasize the potential utility of miRNA testing as a valuable and precise tool for differentiation between HCC patients and individuals with LC. Future research efforts should focus on further validating and extending these findings within this specific context. If confirmed, the incorporation of miRNA testing into clinical protocols could represent a non-invasive and effective strategy for enhancing diagnostic accuracy in distinguishing between HCC and LC, facilitating more targeted and timely interventions in these two patient groups.

This meta-analysis offers numerous advantages. Firstly, it reveals that circulating miRNAs possess significant diagnostic potential in effectively distinguishing between HCC and LC patients. This discovery introduces a fresh outlook on the creation of biomarkers for the differentiation of HCC from LC. Secondly, the meta-analysis undertook a thorough assessment of miRNAs, incorporating subgroup analysis and regression analysis to examine influencing factors like ethnicity, sample source, regulation mode, miRNA profiling, sample size, presence of cut-off values, and various types of HCC. This comprehensive approach aimed to analyze and elucidate the origins of heterogeneity in the findings.

However, it is essential to recognize the limitations of this study. Firstly, variations in the cut-off parameters for miRNAs and internal reference controls across the included studies could serve as a potential source of heterogeneity. Secondly, the meta-analysis did not assess the distinctions in the diagnostic accuracy of miRNAs specifically in HCC cases with diverse clinicopathological features. Thirdly, the ethnicities of the participants were predominantly limited to Asian, European, and African populations. While the identified miRNAs demonstrated remarkable diagnostic value for distinguishing HCC from LC within these ethnic groups, their diagnostic performance may not be universally applicable to HCC patients worldwide. Fourthly, the lack of a substantial number of comparable miRNAs for pooling results hinders the identification of specific single miRNAs or a panel as the optimal diagnostic biomarkers for distinguishing HCC from LC. Therefore, it is crucial to interpret these findings with caution.

In conclusion, this meta-analysis offers evidence supporting the identification of circulating miRNAs as innovative and valuable biomarkers for distinguishing between HCC and LC patients. The detection of miRNAs, especially the upregulated ones, can be employed to distinguish HCC patients from LC. Nevertheless, it is imperative to conduct thorough functional assessments and additional prospective studies involving larger sample sizes and diverse ethnic groups to validate and expand upon these findings.

## Data availability statement

The original contributions presented in the study are included in the article/Supplementary material, further inquiries can be directed to the corresponding author.

## Author contributions

EA: Writing – review & editing, Writing – original draft, Visualization, Validation, Supervision, Software, Project administration, Methodology, Investigation, Formal analysis, Data curation, Conceptualization. MB: Writing – review & editing, Writing – original draft, Visualization, Validation, Supervision, Software, Project administration, Methodology, Investigation, Formal analysis, Data curation, Conceptualization. MW: Writing – review & editing, Visualization, Validation, Supervision, Software, Project administration, Methodology, Investigation, Formal analysis, Data curation. FG: Writing – review & editing, Visualization, Validation, Supervision, Software, Project administration, Methodology, Investigation, Formal analysis, Data curation. ZM: Writing – review & editing, Visualization, Validation, Supervision, Software, Project administration, Methodology, Investigation, Formal analysis, Data curation. MT: Writing – review & editing, Visualization, Validation, Supervision, Software, Project administration, Methodology, Investigation, Formal analysis, Data curation. DA: Writing – review & editing, Visualization, Validation, Supervision, Software, Project administration, Methodology, Investigation, Formal analysis, Data curation. DW: Writing – review & editing, Visualization, Validation, Supervision, Software, Project administration, Methodology, Investigation, Formal analysis, Data curation. AG: Writing – review & editing, Visualization, Validation, Supervision, Software, Project administration, Methodology, Investigation, Formal analysis, Data curation. HE: Writing – review & editing, Visualization, Validation, Supervision, Software, Project administration, Methodology, Investigation, Formal analysis, Data curation.
