# Supplementary material for: Diagnostic accuracy of circulating miRNAs to discriminate hepatocellular carcinoma from liver cirrhosis: a systematic review and meta-analysis
Source: Front Med (Lausanne). 2024 Apr 24;11:1359414. doi: 10.3389/fmed.2024.1359414 (PMC11076824; doi:10.3389/fmed.2024.1359414)
Supplement: Supplementary file 2 [file Table_2.DOCX]

| **SN** | **Database** | **Search terms** | **Articles** | **date** |
| --- | --- | --- | --- | --- |
| 1 | Scopus | (TITLE-ABS-KEY (“plasma microRNAs") OR TITLE-ABS-KEY ("plasma microRNA") OR TITLE-ABS-KEY ("plasma miR*") OR TITLE-ABS-KEY ("serum miRNAs") OR TITLE-ABS-KEY ("serum microRNAs") OR TITLE-ABS-KEY ("serum microRNA") OR TITLE-ABS-KEY ("serum miR*") AND TITLE-ABS-KEY (“diagnos*”) AND TITLE-ABS-KEY ("hepatocellular carcinoma") OR TITLE-ABS-KEY (hcc)) | 170 | 10/08/2023 |
| 2 | Embase | ('circulating mirnas':ti,ab,kw OR 'circulating micrornas':ti,ab,kw OR 'circulating microrna':ti,ab,kw OR 'circulating mir*':ti,ab,kw OR 'plasma mirnas':ti,ab,kw OR 'plasma micrornas':ti,ab,kw OR 'plasma microrna':ti,ab,kw OR 'plasma mir*':ti,ab,kw OR 'serum mirnas':ti,ab,kw OR 'serum micrornas':ti,ab,kw OR 'serum microrna':ti,ab,kw OR 'serum mir*':ti,ab,kw) AND ('Diagnos*':ti,ab,kw) AND ('hepatocellular carcinoma':ti,ab,kw OR 'hcc':ti,ab,kw) | 313 | 10/08/2023 |
| 3 | Pubmed | (“circulating microRNAs" OR " circulating microRNA" OR " circulating miR*" OR “plasma microRNAs" OR "plasma microRNA" OR "plasma miR*" OR "serum miRNAs" OR "serum microRNAs" OR "serum microRNA" OR "serum miR*") AND (“diagnos*”) AND ("hepatocellular carcinoma" OR hcc) | 251 | 10/08/2023 |
| 4 | ScienceDirect | ("miRNAs" OR "miRNA" OR "miR ") AND ("diagnosis") AND ("hepatocellular carcinoma" OR "HCC") | 75 | 10/08/2023 |
| 5 | Wiley online library | ("miRNAs" OR "miRNA" OR "miR") AND ("diagnosis") AND ("hepatocellular carcinoma" OR "HCC") | 32 | 10/08/2023 |
| 6 | Other sources | Utilize the titles that were discovered from the reference lists of articles chosen during electronic database searches. | 3 | 10/08/2023 |

**Searching strategy for diagnostic accuracy of circulating miRNAs to discriminate hepatocellular carcinoma from liver cirrhosis: A systematic review and meta-analysis**
